# Characterization of cervical cancer stem cell-like cells: phenotyping, stemness, and human papilloma virus co-receptor expression

**DOI:** 10.18632/oncotarget.8218

**Published:** 2016-03-20

**Authors:** Elizabeth Ortiz-Sánchez, Luz Santiago-López, Verónica B. Cruz-Domínguez, Mariel E. Toledo-Guzmán, Daniel Hernández-Cueto, Saé Muñiz-Hernández, Efraín Garrido, David Cantú De León, Alejandro García-Carrancá

**Affiliations:** ^1^ Subdirección de Investigación Básica, Instituto Nacional de Cancerología, Secretaría de Salud (SS), México City, Mexico; ^2^ Laboratorio de Marcadores Moleculares, Hospital Infantil de México “Federico Gómez”, SA, Mexico City, Mexico; ^3^ Departamento de Genética y Biología Molecular, Centro de Investigación y de Estudios Avanzados del Instituto Politécnico Nacional (CINVESTAV-IPN), Mexico City, Mexico; ^4^ Subdirección de Investigación Clínica, Instituto Nacional de Cancerología, Secretaría de Salud (SS), México City, Mexico; ^5^ Unidad de Investigación Biomédica en Cáncer, Instituto de Investigaciones Biomédicas, Universidad Nacional Autónoma de México (UNAM) and Instituto Nacional de Cancerología, Secretaría de Salud (SS), Mexico City, Mexico

**Keywords:** cervical cancer, cervospheres, cervical cancer stem cell phenotype, stemness markers, ALDH activity

## Abstract

Cancer stem cells (CSC) exhibit high tumorigenic capacity in several tumor models. We have now determined an extended phenotype for cervical cancer stem cells. Our results showed increased CK-17, p63^+^, AII^+^, CD49f^+^ expression in these cells, together with higher Aldehyde dehydrogenase (ALDH^bright^)activity in Cervical CSC (CCSC) enriched in cervospheres. An increase in stem cell markers, represented by OCT-4, Nanog, and β-catenin proteins, was also observed, indicating that under our culture conditions, CCSC are enriched in cervospheres, as compared to monolayer cultures. In addition, we were able to show that an increased ALDH^bright^ activity correlated with higher tumorigenic activity. Flow cytometry and immunflorescence assays demonstrated that CCSC in cervosphere cultures contain a sub-population of cells that contain Annexin II, a Human papillomavirus (HPV) co-receptor. Taken together, under our conditions there is an increase in the number of CCSC in cervosphere cultures which exhibit the following phenotype: CK-17, p63^+^, AII^+^, CD49f^+^ and high ALDH activity, which in turn correlates with higher tumorigenicity. The presence of Annexin II and CD49f in CCSC opens the possibility that normal cervical stem cells could be the initial target of infection by high risk HPV.

## INTRODUCTION

Cervical cancer (CC) continues to be an important human public health problem in developing countries [1, 2]. There are some ambulatory surgical procedures, for example, cryosurgery and electro-surgery to remove and cure premalignant lesions. However, for high-risk premalignant lesions and carcinomas, aggressive protocols are the therapeutic options for patients, including chemo- and radiotherapies (reviewed in [3]). Furthermore, the majority of patients with CC exhibit tumor recurrence after treatment, a phenomenon that could be explained, in part, by the hierarchy theory of carcinogenesis, in which only Cancer Stem Cells (CSC) possess the capability to promote and support tumor growth [4]. Additionally, disease relapse could also be a consequence of resistant cancer cell clonal selection, including stem and/or non-stem cells, in which the accumulation of mutations in these cells can be associated with the ability to develop anti-cancer therapy resistance resulting in tumor progression [5]. These CSC can bypass drug cytotoxicity due to the presence of an efflux pump belonging to the Adenosine triphosphate (ATP)-dependent protein family, such as ABCG2, which has been observed to be increased in several CSC [6]. Additionally, CSC can promote anti-apoptotic mechanisms to prevent drug effects.

In addition to quiescence or the resting G0 cell-cycle state, CSC share phenotype surface markers with their normal counterparts [7-9]. However, because normal cervical epithelial stem cell markers remain unknown, general strategies are suggested to isolate CSC-enriched subsets and early progenitors, such as Side population (SP) and Aldehyde dehydrogenase activity (ALDH) assays. Villanueva and collaborators have reported the presence of a SP in SiHa and CaLo cervical cancer cell lines, in which these SP cells have shown properties of CSC, such as the capacity to form colonies in clonogenic assays [10]. In our group, HeLa SP and CD49f (α-integrin) cells were also evaluated in HeLa cervospheres [11]. ALDH activity has also been used to identify CSC. It has been reported that ALDH activity is related to drug detoxification by aldehyde oxidation, which in turn is related to chemo and radioresistance of CSC and to the maintenance of the CSC population. However, the role of this enzyme in stemness remains unknown. There are some reports that demonstrate that ALDH activity is related to an increase of Hypoxia transcription factor HIF-2α expression. This transcription factor is related with the expression of OCT-4, a stem cell transcription factor necessary for maintaining a stemness state (reviewed in [12]).

Indeed, it has been observed that cells with high ALDH activity are able to induce greater tumor growth compared to ALDH-negative subpopulations, thus, high ALDH activity evaluation has been employed to identify and to isolate CSC from several tumors, such as ovarian cancer [13], prostate cancer [14], lung cancer [15], breast cancer [16], leukemic stem cell cancer [17], gastrointestinal neuroendocrine tumors [18], head and neck tumors [19], sarcoma [20], and more.

In this work, we established sphere cultures from cervical cell lines (denominated cervospheres) utilizing the commercial epithelial stem cell sphere conditioned medium Mammocult^®^ to enrich the cervical CSC pool through the self-renewal capability of CSC. Using Flow cytometry (FC), we analyzed some phenotype stem-cell markers such as cytokeratine 17 (CK-17), p63 (a homolog of p53 related with embryogenesis), and Annexin II (AII), a protein characterized as a HPV co-receptor. We also demonstrated that our cervospheres showed a stemness state characterized by the presence of OCT-4 and Nanog transcription factors.

Taken together, we demonstrated, to our knowledge for the first time, that the profile of CD49^+^ AII^+^ p63^+^, CK-17^+^ and ALDH^bright^ activity can be considered as a phenotype of putative Cervical cancer stem cells (CCSC). Additionally, because we demonstrated that these cells express AII, a co-receptor necessary for Human papillomavirus (HPV) entry into cells together with Growth Factor Receptor (GFR) and with CD49f (α-integrin), we suggest that these CCSC could be infected by HPV. Finally, latent HPV infections could be explained as part of the resting immune system, and if HPV is able to infect reserve cells, these could be “epithelial stem cells” (in our hypothetical model), present in the epithelial basal layer. This infection may be on standby status, in parallel with the resting state of stem cells, until an event occurs to initiate the malignant transformation program in order to generate CCSC. However, we still lack evidence to confirm that the CCSC can be generated by HPV infection of a normal epithelial stem cell.

## RESULTS

### Phenotype characterization of cervospheres: putative cervical cancer stem cells

**Figure 1:**
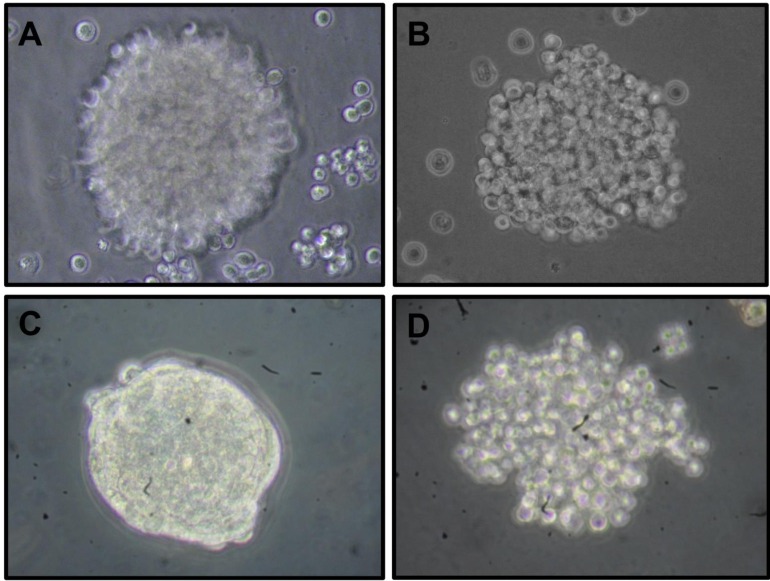
Morphological differences between cervospheres enriched with cancer stem cells derived from human cervical cancer cell lines Optical microscopy images show the morphological differences of cervospheres derived from HeLa **A.** SiHa **B.** Ca Ski **C.** and C-33 A **D.** cell lines cultured in Mammocult ^®^serum-free media under tissue culture conditions for 7 days using a 40X objective (Olympus CK31 microscope).

**Figure 2:**
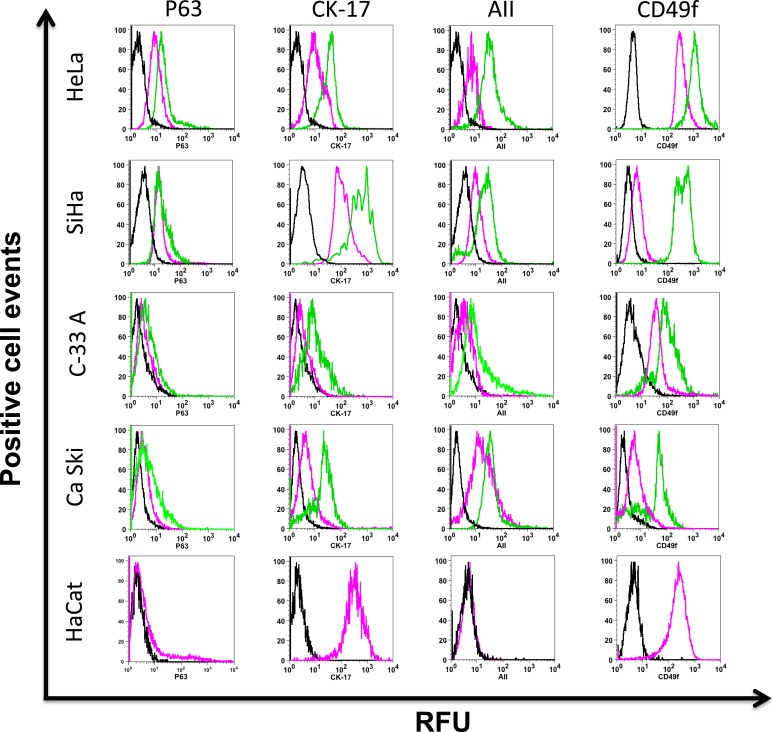
Putative cervical cancer stem cell phenotype present in cervospheres Monolayer (pink line) and cervosphere cells (green line) derived from HeLa, SiHa, Ca Ski, C-33 A and HaCat cell lines were incubated with specific antibodies to detect p63, CK-17, AII and CD49f proteins, using flow cytometry. Dark lines show isotope control. HaCat cell line was used as non-tumorigenic cells control and HaCat cells do not form spheres in our cell culture conditions. Data is representative of at least three independent experiments. Ten thousand cells are recorded for their analysis using the FloJo ^®^ software. RFU (Relative fluorescence units)

HeLa (HPV-18), SiHa (HPV-16), Ca Ski (HPV-16) and C-33 A (HPV-negative) cell lines were cultured in Mammocult^®^ to promote self-renewal of the CCSC present in cell lines, as well as to maintain their dedifferentiated state. The cervospheres are depicted in Figure 1. Different cervosphere morphologies can be observed, which could be related to the differences among the cell lines tested. To characterize the cells that make up the cervospheres, we analyzed the presence of p63, Cytokeratin-17 (CK-17), and Annexin II (AII). In Figure 2, a discrete increase of p63 protein was observed in cells from HeLa cervospheres (Mean Florescence Intensity [MFI] = 20.1±2.5 relative fluorescence units [RFU]) compared to their monolayer counterpart cells (MFI = 8.9±2.0 RFU). It has been demonstrated that p63 is involved in morphogenesis and has been proposed as a stem cell marker for the epithelial cervix [21]. However, under our conditions, the p63 protein did not increase after SiHa, Ca Ski and C-33 A cervosphere formation as the MFIs in these cases are almost the same (ie: in C-33 A monolayer MFI = 2.36±1.3 RFU compared to MFI = 4.41±1.5 RFU in sphere condition). Additionally, CK-17 has been suggested as a cervical stem cell marker [21, 22]. The majority of our monolayer cells are CK-17^+^ (except C-33 A); however, there was an increase of CK-17 detection in all cervosphere cells tested, which could be related with CCSC-like enrichment. Interestingly, similar to CD49f expression in cervical stem cell-like cells, tested previously [11], AII is another HPV co-receptor found to be increased in HPV-infected cervosphere cells but less in C-33 A cells, a negative HPV CC cell line and in HaCaT cells, a non-tumorigenic immortalized cell line.

### Stemness markers are present in cervospheres, a cell culture enriched in cervical stem cells

**Figure 3:**
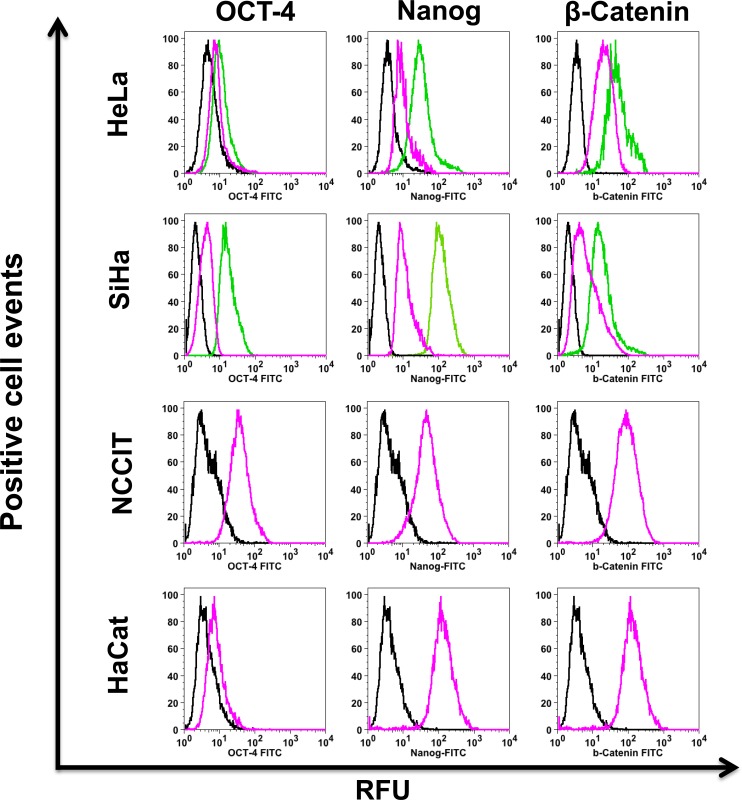
Stemness markers are increased in cervosphere cells Specific OCT-4, Nanog and β-catenin antibodies were used to detect the stemness markers present in the cervospheres enriched in cervical cancer stem cells by flow cytometry. Data are representative of three independent experiments. Pink and green lines represent the assays in monolayer and cervosphere cells, respectively. Black lines show Isotype controls. Ten thousand cells are recorded in BD FAC Scan^TM^ and then analyzed in FloJo ^®^ software. RFU (Relative fluorescence units)

Transcription factors OCT-4 and Nanog are conventional markers used to demonstrate cell stemness. Figure 3 shows that there is an increase of Nanog protein detected in HeLa and SiHa cervospheres, compared with their monolayer cells. In the case of OCT-4 protein, its increase was clearly detected in SiHa cervospheres, and there was a discreet increase of OCT-4 detected in HeLa cervosphere cells. This is an expected result as data published by the Schöler group in 2008, demonstrated that HeLa monolayer cells lack OCT-4 messenger RNA (mRNA) and protein expression [23]. However, this tiny increase could be due to the sphere stem cell-like tissue culture condition, in that we also were able to detect OCT-4 mRNA in HeLa cervospheres by Q-RT-PCR (Supplementary Figure 1).

Since the Wnt/β-catenin cell signaling pathway increases Nanog expression [24], we evaluated β-catenin protein in the cervospheres. Figure 3 shows that β-catenin protein is clearly increased in SiHa cervospheres compared to monolayer counterpart cells (MFI = 17.4±2.3 and 6.4±1.4 RFU, respectively). In addition, β-catenin was also increased to a lesser extended in HeLa cervospheres compared to HeLa cells cultured under monolayer tissue culture conditions (MFI = 28±1.1 and 22.6±1.3 RFU, respectively). The teratocarcinoma NCCIT cell line was used as positive control for stemness markers.

### ALDH activity was increased in cervospheres, another cervical stem cell-like property and phenotype

**Figure 4:**
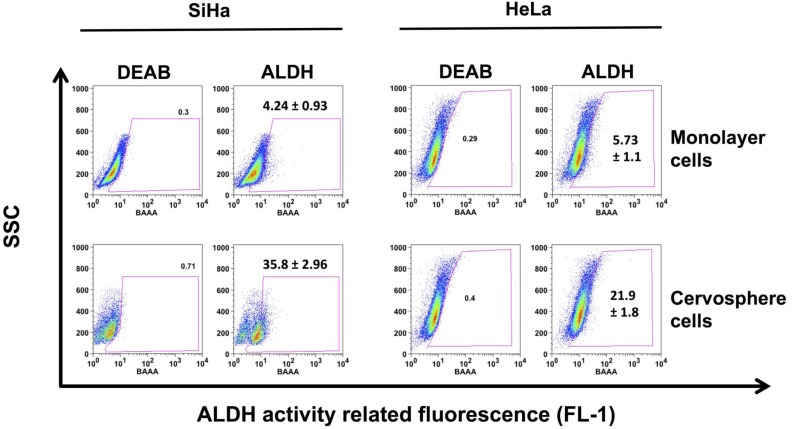
ALDH activity is increased in cervical cancer stem cell enriched cultures The ALDEFLUOR kit^®^ was used to evaluate the percentage of cells derived from HeLa and SiHa cervospheres compared to their monolayer counterparts. Dot blots are representative of at least three independent assays. Ten thousand cells are recorded in BD FAC-Scan^TM^ and then analyzed in FloJo ^®^ software.

In addition to evaluating putative stem cell markers such as p63, CK-17, and AII proteins, we determined ALDH activity in these cells. Figure 4 shows an increase of Aldehyde dehydrogenase (ALDH) activity in HeLa and SiHa cervosphere cells, indicated by the percentage of cells that showed intensification of fluorescence intensity (21.9±1.8 % and 35.8±2.96 %, respectively) compared to their monolayer counterparts (5.73±1.1% and 4.24±0.93%, respectively).

Specifically, in SiHa cervospheres, a subpopulation is clearly shifted to the right and two ALDH- positive subpopulations: ALDH^bright^ and ALDH^low^ are observed. Interestingly, the ALDH^bright^ cells derived from HeLa and SiHa cervospheres are more tumorigenic compared to cells originating from whole spheres (see Table 1), as tested by *in vivo* assays. The tumor development after challenge with 10,000 ALDH^bright^ cells was faster and greater compared with xenotransplants derived from monolayer cells. Actually, SiHa ALDH^bright^ cell tumors grow faster than HeLa ALDH^bright^ cell tumors. In contrast, HeLa ALDH^brigth^ cell tumors were larger than ALDH^bright^ tumors derived from SiHa cervospheres (data not shown). Thus, all results demonstrate, once again, that the CC stem/tumor initiating cell phenotype includes an increase in ALDH enzyme activity.

**Table 1:** Tumorigenic capability of cervical cancer stem cell-like cells in *nu/nu* mice

	HeLa				SiHa			
# cells	Monolayer	Sphere	ALDH+	ALDH-	Monolayer	Sphere	ALDH+	ALDH-
**1×10^3^**	0/5	0/5	3/5	0/5	0/5	0/5	4/5	0/5
**1×10^4^**	0/5	5/5	5/5	0/5	0/5	4/5*	5/5	0/5
**1×10^5^**	0/5**	5/5***	NT	NT	0/5	5/5**	NT	NT

ALDH+ (ALDH^bright^ cells: high ADLH activity)

ALDH− (ALDH^low^ cells: poor ALDH activity)

NT: not tested

* Tumor growing (≈ 0.5 cm) was observed 30 days after inoculation.

** Tumor growing (≈ 0.5 cm) was observed in 1/5 mice 60 days after inoculation.

*** Tumor growing (≈ 1 cm) was observed 21 days after inoculation.

### Annexin II, another HPV co-receptor present in cervosphere cells with high tumorigenic capability

**Figure 5:**
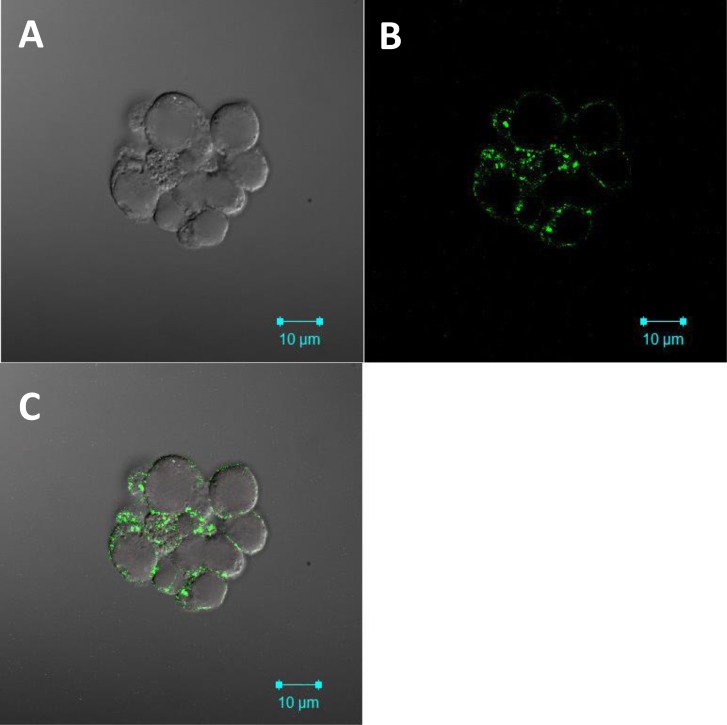
Annexin II HPV co-receptor is expressed on the cell surface of cervosphere cells SiHa cervospheres were fixed and paraffin embedded. Four μm sections were incubated with specific AII antibodies (green). Confocal images were taken under 100 X objectives. **A.** Nomarski image, **B.** Annexin II detection (green color) and **C.** Merge. Axiovision Image was used for image acquisition. Scale bars, 10 μm.

Because CD49f is a co-receptor that is increased in cervosphere cells, it was important to determine whether these cells derived from SiHa cervopheres showed an increase of HPV AII co-receptor on their surface (Figure 2). In Figure 5, AII was clearly and specifically detected on the surface of cervosphere cells with high tumorigenic capability (Table 1), using confocal microscopy. This confocal analysis demonstrated, to our knowledge for the first time, that the cervical cancer stem/tumor initiating cell-like cells contain the AII HPV co-receptor on their surface, making them target cells for HPV entry and infection (Figure 5). Moreover, we also evaluated the presence of p63, CK-17, and AII proteins on cells derived from biopsies of patients with CC. Interestingly, in a tissue sample of a patient with a benign lesion, AII protein was undetected (Supplementary Figure 2).

## DISCUSSION

Since we studied cells with stemness and putative stem cell markers in cervical cancer cell lines growing in monolayer tissue culture conditions, it is necessary to claim for them the name stemloids, a term used to describe proliferating cells with self-renewal capacity [9]. This term emphasizes the absence of quiescence cells (stem) in proliferating cell lines. Villanueva and colaborators showed the presence of a G_0_ subpopulation of cells (quiescence) in a side population derived from the HeLa cell line, additionally demonstrating their self-renewal capacity as shown by an increase in side population cells after serial sorting assays [10]. We must be careful to use the term cervical cancer stem cells (CCSC), however, since our spheres are cultured without fetal bovine serum (which contains differentiation stimuli) and we using a commercial medium for enriching stem cells with EGF and bFGF, thus, we suggest that our spheres are enriched for cervical tumorigenic cells and they could named cervical cancer stem cell-like cells, characterized by stemness and cervical stem markers. Therefore, in this work we propose an extended putative CCSC phenotype.

In addition to CD44 and CD133, previously studied by other authors under different models, we include the detection of CD49f, CK-17, p63 and AII proteins as putative CCSC markers in enriched cultures. Mazuko and collaborators demonstrated, *in vivo,* a protective effect of inhibiting the stem cell receptor CD44RI using an antibody against this isoform, in mice previously challenged with 1×10^6^ cells from ME180, a human cervical cancer cell line [25]. Furthermore, Feng and collaborators evaluated the presence of CD44 and CK-17 in tumorigenic cervospheres injected into mice challenged with 100,000 total cervosphere cells [26]. In contrast, the tumorigenicity of cervospheres grown under our conditions show tumor growth capability using 10,000 cells, a small amount compared to previous reports, suggesting that our cervospheres are a CCSC enriched culture compared to monolayer cells.

Initially, in the sphere formation assay, we can observe that the four cervical cell lines tested possess the capacity to form cervospheres. However, these spheres show, to a certain degree, different morphology, size, and compactness (Figure 1). It was clearly shown that Ca Ski cervospheres are more compact compared with other cervospheres. Furthermore, HeLa, SiHa, and C-33 A cervospheres demonstrated a relaxed morphology under our conditions. These disparate morphologies could be related with their different cell line origin. Thus, HeLa are derived from HPV-18 infected adenocarcinoma, Ca Ski and SiHa are HPV-16 infected squamous carcinoma, and C-33 A is a HPV-negative carcinoma. By testing HeLa and SiHa cervospheres using a cell line passage number less than 15, we observed discrete morphological changes. Furthermore, the cervosphere compactness could be cell line origin associated as well (data not shown).

In our cervospheres, we demonstrated that there are increases in p63, CK-17, and Annexin II (AII) proteins in cells belonging to HeLa, SiHa, Ca Ski, and C-33 A cervospheres compared with their monolayer counterparts (Figure 2). The p63 and CK-17 proteins have been considered putative normal cervical stem cell markers [21] due to their role in morphogenesis. P63 is a transcription factor belonging to the p53 family of normal epithelial stem cells and its role has been demonstrated in maintaining the immature epithelial state in endometrium cells, and progenitor cells of breast and cervical epithelia [27]. Specifically, ΔNp63 is an isoform that lacks a p53-like transactivation domain, usually expressed in squamous and glandular epithelial tissues, where it is involved in stem cell renewal. Employing a mammary stem cell model using serial sphere-formation assays, it was demonstrated that Np63 is a key protein related to self-renewal [28]. Furthermore, it was demonstrated that p63 is also implicated in the Sonic-Hg signaling pathway, in a manner that is likely stemness-responsible, through the induction of Bmi-1, a protein necessary for stem-cell proliferation [28-30].

Additionally, we observed an increase in cytokeratin 17, which has also been implicated as an epithelial stem cell marker [21]. Using immunohistochemical analysis, CK-17 was detected in a small number of cells located in the basal layer, additionally, CK-17 expression was increased in parallel with high, premalignant and cancer lesions. Remarkably, CK-17 negative premalignant specimens were obtained from patients who did not progress to CC [31], while patients with CK-17 positive cells had a greater probability of progression, suggesting its relevance in cervical carcinogenesis. Recently, 1,750 patients with different grades of premalignant and cancer lesions were included in a study in which the authors validated the use of CK-17 positive cells as a poor prognostic marker [32].

Martens and collaborators in 2009 suggested the presence of two subpopulations of reserve cells related to the expression of p63 and CK-17. CK-17 and p63positive cells are progenitor cells that give rise to endo- and ecto-cervical epithelial cells. In contrast, CK-17 negative cells are reserve cells from which only endocervical epithelial cells arise [33]. CK-17 positive cells were also detected in immature squamous metaplasia [34]. Therefore, our data make a contribution of putative CCSC markers for future studies, enhancing previous publications studying CD44 expression and ALDH activity. In this work, these proteins were also assessed with CD49f and Annexin II protein detection.

The first contact between HPV and the host cell is initiated by heparan sulfates and glycosaminoglycans. On the surface, after HPV interaction with CD49f (an alpha-integrin), a subsequent HPV Annexin II co-receptor is required for full virus-cell interaction. Furthermore, HPV is coated with Clathrin, caveolin, and cholesterol to engender dynamic endocytosis. In the cytosol, the viral genome is released to continue the viral cell cycle [35]. In this work, we demonstrated the presence of both HPV co-receptors in the subpopulation enriched with CCSC-like cells. First, we observed a vast presence of CD49f in cells cultured under non-stem cell conditions in the presence of fetal bovine serum (FBS) (Figure 2). Furthermore, an increase of CD49f positive cells with more surface expression of CD49f molecules was observed in cervospheres, in which the subpopulation of cancer stem cell-like cells is enriched, compared to monolayer cultures. This was also confirmed by tumorigenic *in vivo* assays (Table 1). It has been discussed that the poor ability of tumor-sustaining cells can be a consequence of the limited capability of human cancer cells to proliferate in foreign microenvironments (reviewed in [9]. However, we observed that a small amount of ALDH^bright^ cells are able to induce tumor growth compared to monolayer cells, indicating that these cells adapted in the nude mice microenvironment.

Interestingly, in addition to the increase of CD49f, we observed a clear increase of Annexin II (AII), the other HPV co-receptor, in the CCSC-like enriched sphere cultures from SiHa, Ca Ski (HPV16), and HeLa (HPV18) cells, in comparison to monolayer cultures (Figure 2). In contrast, the presence of AII in C-33 A cells was less compared to the other cancer cells. These data were confirmed by confocal images which illustrate the presence of AII HPV co-receptor on the surface of the CCSC-like that are enriched in our cervospheres. The phenotype marker assay was also tested in CC biopsies. Interestingly, CK-17, p63, and CD49f proteins were detected in cells derived from tumor biopsies. However, AII protein was present only in malignant cervical lesions and not in benign lesions, suggesting that AII could be a key co-receptor for HPV cell infection, including cervical stem cells (Supplementary Figure 2).

In addition to FC assays, the presence of HPV AII receptor on the surface of CCSC-like cells was also shown by immunofluorescence, suggesting that normal cervical cells, as part of the reserve cells of the cervical epithelium, are able to be infected by HPV (Figure 5). However, we are unable to suggest that HPV infection converts normal cervical stem cells into CC stem cells.

In order to characterize the cells that make up the cervospheres tested, stemness markers were also detected by FC assay. For the specific cases of HeLa and SiHa cervospheres, an increase of transcription factors OCT-4 and Nanog was detected. We suggest that the disparate-detection level of these stemness markers in HeLa and SiHa cells can be related to their different cell origin (deriving from simple and squamous epithelia, respectively) (Figure 3). The β-catenin protein is also related to the self-renewal capacity of stem cells due to its role in the Wnt/β-catenin cell signaling pathway. The target genes of β-catenin are involved in OCT-4 transcription, a key protein for the transcription of Nanog and SOX-2 required for self-renewal and for maintaining the undifferentiated state. It is interesting to observe that the greatest total β-catenin detected correlated with the higher OCT-4 protein in SiHa cervosphere cells compared to HeLa cervosphere cells (Figure 3).

Continuing with CCSC-like characterization, the high activity of the ALDH enzyme employed for isolating cancer stem cells in several tumors was also evaluated in SiHa and HeLa cervospheres, demonstrating an increase in an ALDH^bright^ subpopulation in this enriched stem cell-conditioned culture compare to monolayer cell cultures (Figure 4). Due to this high ALDH activity, these cells were sorted and injected subcutaneously (*s.c.)* into nude mice. The *in vivo* assay demonstrated that cells with high ALDH activity (Table 1) are more tumorigenic compared to ALDH^dim^ cells, as was also observed by the Liu group [36]. This data suggested that CCSC are present in the ALDH^bright^ subpopulation, and it is a marker that can be used for target therapy.

Several leading authors in CSC, stress the need for specific chemotherapies to eliminate CSC in patients. The conventional chemotherapy kills proliferating cancer cells and not cancer stem cells; there is possible clone selection of a resistant proliferating cancer cell with enough proliferation capabilities to promote disease recurrence and kill a patient [5, 9]. A combination therapy must be designed to eliminate cancer proliferating cells, therapy-resistant cancer cells and also cancer stem cells. It is known that normal and cancer stem cells are identical except for their tumorigenic capacity; therefore greater efforts are needed for genomic, epigenetic and proteomic studies to identify a specific gene, protein or pathway present only in cancer stem cells and not in normal stem cells.

In summary, we have suggested an additional phenotype for CCSC: CD49f^+^, AII^+^, CK-17^+^ p63^+^ ALDH^bright^. Furthermore, it is clear that the behavior and/or phenotype could be different between cancer stem cells from adenocarcinomas and those from squamous carcinomas. Because HPV-16 and HPV-18 exhibit a specific difference in gene expression regulation, we are able to suggest that the CCSC present in these cultures are also different. Taken together, we demonstrated that CCSC-like cells are positive for both HPV receptors, CD49f and for AII, suggesting that cervical stem cells could have been HPV-infected and may be responsible for the origin of the cervical cancer and maintaining tumor growth.

## MATERIALS AND METHODS

### Tissue culture

Human CC cell lines, HeLa (adenocarcinoma, HPV-18) and SiHa (squamous cell carcinoma, HPV-16) were purchased from ATCC (American Type Culture Collection, Manassas, VA, USA). Ca Ski (epidermoid carcinoma HPV-16), and C-33 A (carcinoma, HPV-negative) cervical cell lines and the NCCIT teratocarcinoma cell line were kindly donated by Dr. Alejandro García-Carrancá (Instituto Nacional de Cancerología, Mexico City), while the HaCaT (non-tumorigenic skin keratinocyte) cell line was kindly donated by Dr. Nobert Fusenig. These cell lines were authenticated by Laboratorio de Diagnóstico Genómico at Instituto Nacional de Medicina Genómica and by University of Colorado Cancer Center. NCCIT teratocarcinoma cells were used as positive control for stemness markers. Cells were cultured in DMEM media (Life Technologies Corporation, Carlsbad, CA, USA) supplemented with 10% (v/v) FBS, 50 U/mL Penicillin, and 50 μg/mL Streptomycin. Cells were cultured at 37°C, 5% CO_2_. Finally, HeLa and SiHa cell lines under 25-cell passage were employed for the assays.

### Cervospheres

Prior to initiating the sphere culture derived from CC cell lines (cervospheres), monolayer cells must be found in healthy condition at 70–80% confluence. These cells were harvested, counted, and washed with Phosphate buffer solution (PBS) to remove the remainder of FBS. One thousand cells per mL of Mammocult^®^ medium (Stem Cell Technologies, Vancouver, BC, Canada) were seeded in ultra-low adherence dishes and 6-well plates (Corning, Inc., Corning, NY, USA). Cells were cultured under tissue culture conditions for 7 days. Sphere formation was monitored daily.

### Flow cytometry

#### Phenotyping assays

Cervospheres were collected and placed in a 15-mL tube, where they were allowed to remain for 10 min. After some time had elapsed, supernatant was removed and the bottom sphere cells were washed with PBS and collected as previously mentioned. Pelleted cervospheres were suspended in PBS and disaggregated by mechanic pipetting. For each primary antibody, 5 × 10^5^ cells were incubated with anti-p63, anti-CK-17, Annexin II (AII) (all of these by Santa Cruz Biotechnology, Inc., Dallas, TX, USA) in flow buffer (1X PBS, 0.05% BSA) on ice. After 30 min, cells were washed with flow buffer and spun down at 500 *g* (*r* = 11 cm) for 5 min at room temperature. Then, cells were incubated with FITC-coupled secondary antibody for 30 min on ice. After some time had elapsed, cells were once again washed in flow buffer and fixed with 4% p-formaldehyde in PBS. For CD49f detection, 5 × 10^5^ cells were incubated with anti-CD49f-PE (BD Bioscience, CA, USA) on ice for 30 min. Then, cells were washed with flow buffer and fixed. Cells were also incubated with isotype controls. Staining cells were read in BD FAC-Scan™ (BD Bioscience). At least, ten thousand events were recorded for each flow cytometer measurement. FlowJo^®^ software was utilized for analyzing data.

### Stemness markers

Cells from monolayer and cervospheres were collected, washed, and counted. For antibody- treatment incubation, 5 × 10^5^ cells were permeabilized by incubation with methanol for 15 min on ice. Then, cells were washed with flow buffer and incubated with primary antibody anti-OCT-4, Nanog, and β-catenin. After 30 min of incubation on ice, cells were washed and then incubated with the secondary antibody FITC-conjugated for an additional 30 min on ice. Finally, cells were washed and suspended in 4% p-formaldehyde, end-reading them employing BD FAC-Scan^TM^ flow cytometry (BD Bioscience). Then, at least 10,000 events were recorded for each FC measurement.

### ALDH activity

ALDH activity was evaluated by using the ALDEFLUOR kit^®^ (Stem Cell Technologies). Briefly, cells were harvested, washed, counted, and suspended in ALDEFLUOR buffer at a density of 1 × 10^6^ cells/mL. Cells were incubated with 1.5 μM ALDEFLUOR substrate. One half was co-incubated with the ALDH inhibitor DEAB. Both conditions were incubated at 37°C in a water bath. After 45 min, cells were spin down and suspended in ALDEFLUOR buffer. Cells were acquired in a BD FAC-Scan cytometer^TM^. 10,000 events were recorded. For cell sorting, BD FAC-SAria II was used and cells were recovered under serum replacement to maintain their viability for *in vivo* assays.

### Immunofluorescence

Sphere cells were fixed with 4% formaldehyde, dehydrated, and paraffin-embedded. Serial cuts of 4 μm were obtained for immunofluorescence assays. Briefly, the cells were rehydrated in decreasing concentrations of xylol-ethanol, and antigens were released with citrate buffer and washed in PBS. Then, sphere cuts were blocked in 1% albumin solution for 30 min at room temperature. After washing in PBS, samples were incubated with the primary antibody for 2 h, were washed, and were further incubated with the secondary antibody for 1 h (Alexa Fluor 480 or 568 according to the case). Sections were then washed and laid on Vectashield mounting medium (Vector Lab, USA) to be observed under confocal microscopy (LSM 5 Carl Zeiss México) under 100X objective. Axiovision Image Software was used for software.

### *In vivo* tumorigenic assays

BALB/c *nu/nu* female mice were used in this work to test the tumorigenic capacity of CCSC-like enriched cultures. These animals were under 4–6 weeks of age, and were obtained from the Instituto Nacional de Ciencias Médicas y Nutrición “Salvador Zubirán” (INNSZ) (“Salvador Zubirán” National Institute for Medical Sciences and Nutrition). Mice were xenotransplanted subcutaneously** (s.c). with HeLa and SiHa monolayer cells and their respective cervosphere cells at different amounts, using six mice per group. Additionally, other mice groups were challenged via subcutaneously (s.c) with previously sorted ALDH high-activity cells. Mice were monitored for at least 3 months. For subcutaneous tumors in mice, the maximal allowable size is 2 cm in diameter (Tumor Policy for Mice and Rats from Boston University Research Compliance).

### Statistical analysis

Data are represented as the mean ± Standard deviations (SD) of at least three independent experiments. The Student *t* test was used to determine statistical significance (*p* < 0.05) using Microsoft Excel 2011.

## SUPPLEMENTARY FIGURES


