# Supplementary material for: Characterization of cervical cancer stem cell-like cells: phenotyping, stemness, and human papilloma virus co-receptor expression
Source: Oncotarget. 2016 Mar 20;7(22):31943–54. doi: 10.18632/oncotarget.8218 (PMC5077987; doi:10.18632/oncotarget.8218)
Supplement: Supplementary file 1 [file oncotarget-07-31943-s001.pdf]

Characterization of cervical cancer stem cell-like cells: phenotyping, stemness, and Human Papilloma Virus co-receptor expression

Supplementary Material

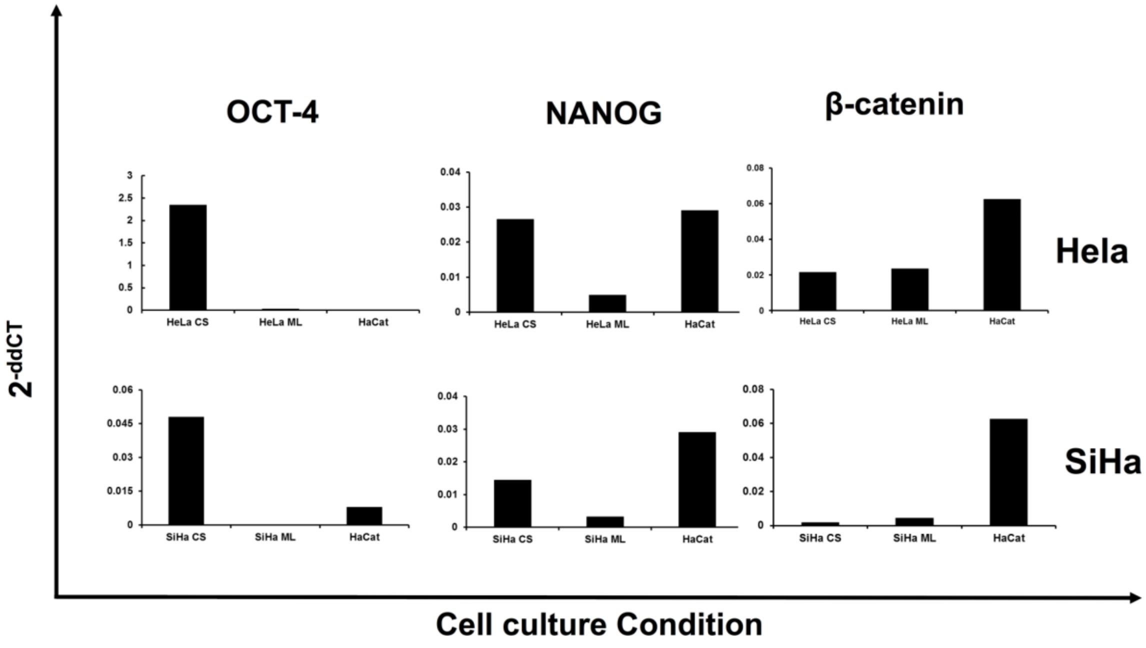

Supplementary Figure 1. Stemness marker overexpression in cervosphere cells by qRT-PCR. mRNA from cervosphere cells (CS) and monolayer cells (ML) were obtained and cleaned to perform real time RT-PCR to evaluate expression of stemness markers OCT-4, NANOG and β-catenin. Data were normalized to GAPDH expression. mRNA from HaCaT cells was used as non-malignant control and the teratocarcinoma NCCIT cell mRNA was used as positive control for stemness marker expression (data not shown). The data is representative of 2 experiments.

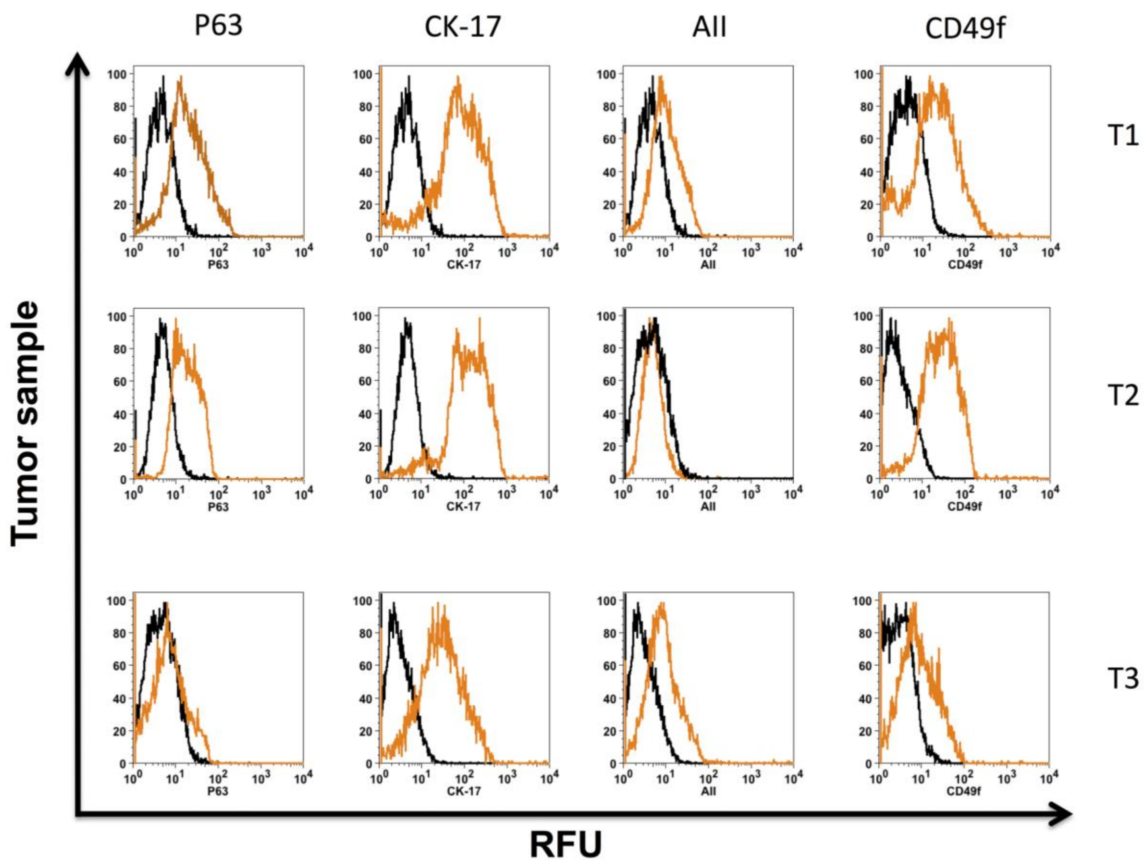

Supplementary Figure 2. Putative cancer stem cell phenotype evaluation in patients with cervical cancer. Samples were obtained in accordance with international ethical standards and guidelines and in compliance with the Declaration of Helsinki and with the approval of the ethics committee of the Instituto Nacional de Cancerología. Three biopsies were obtained after informed consent. Samples were processed and then stained with specific antibodies (orange lines) and their isotype controls (black lines). After pathological analysis, T1 and T3 samples were considered to be cervical cancer. In contrast, pathology testing presented T2 as a benign cervical biopsy. Ten thousand events were recorded for the analysis using the software FloJo®.
